# *In Vitro* Modulator Effect of Total Extract from the Endophytic *Paenibacillus polymyxa* RNC-D in *Leishmania (Leishmania) amazonensis* and Macrophages

**DOI:** 10.1155/2020/8895308

**Published:** 2020-08-27

**Authors:** Débora Meira Neris, Letícia Gonçalves Ortolani, Cynthia Aparecida de Castro, Ricardo de Oliveira Correia, Joice Margareth de Almeida Rodolpho, Luciana Camillo, Camila Tita Nogueira, Cristina Paiva de Sousa, Fernanda de Freitas Anibal

**Affiliations:** ^1^Laboratory of Inflammation and Infectious Diseases, Department of Morphology and Pathology, Federal University of São Carlos, São Carlos, Brazil; ^2^Laboratory of Microbiology and Biomolecules, Federal University of São Carlos, São Carlos, Brazil

## Abstract

Leishmaniases are diseases with high epidemiological relevance and wide geographical distribution. In Brazil, *Leishmania (Leishmania) amazonensis* is related to the tegumentary form of leishmaniasis. The treatment for those diseases is problematic as the available drugs promote adverse effects in patients. Therefore, it is important to find new therapeutic targets. In this regard, one alternative is the study of biomolecules produced by endophytic microorganisms. In this study, the total extract produced by the endophytic *Paenibacillus polymyxa* RNC-D was used to evaluate the leishmanicidal, nitric oxide, and cytokines production using RAW 264.7 macrophages. The results showed that, in the leishmanicidal assay with *L. amazonensis*, EC50 values at the periods of 24 and 48 hours were 0.624 mg/mL and 0.547 mg/mL, respectively. Furthermore, the cells treated with the extract presented approximately 25% of infected cells with an average of 3 amastigotes/cell in the periods of 24 and 48 hours. Regarding the production of cytokines in RAW 264.7 macrophages infected/treated with the extract, a significant increase in TNF-*α* was observed at the periods of 24 and 48 hours in the treated cells. The concentrations of IFN-*γ* and IL-12 showed significant increase in 48 hours. A significant decrease in IL-4 was observed in all cells treated with the extract in 24 hours. It was observed in the treated cells that the NO production by RAW 264.7 macrophages increased between 48 and 72 hours. The endophytic *Paenibacillus polymyxa* RNC-D extract modulates the mediators of inflammation produced by RAW 264.7 macrophages promoting *L. amazonensis* death. The immunomodulatory effects might be a promising target to develop new immunotherapeutic and antileishmanial drugs.

## 1. Introduction

Leishmaniases are infectious diseases caused by the protozoa of *Leishmania* genus. These diseases are neglected and endemic in 98 countries, with higher occurrence in developing countries. Those diseases are associated with malnutrition and precarious housing conditions, among other problems, with about 1 billion people in hazardous areas [1].

Worldwide, there are over 0.2 to 0.4 million new cases of visceral leishmaniasis (VL) and 0.7 to 1.2 million new cases of cutaneous leishmaniasis (CL) every year. It is estimated that 75% of registered cases are concentrated in Brazil, Afghanistan, Algeria, the Islamic Republic of Iran, Pakistan, Peru, Colombia, Saudi Arabia, and the Syrian Arab Republic. Almost 90% of the cases of mucocutaneous leishmaniasis occur in Bolivia, Brazil, and Peru [1].

*Leishmania amazonensis* is one of the species that causes cutaneous leishmaniasis in South America. Widely distributed in Brazil, *L. amazonensis* can lead to development of two forms of the disease: localized cutaneous leishmaniasis and diffuse cutaneous leishmaniasis, characterized by multiple nodules [2].

CL is the most common form of the disease. It is defined by causing ulcers, which can be scattered throughout the body (face, arms, and legs). As a result, it may cause disability and social exclusion [1, 3]. The clinical evolution of CL is intimately related to the immune response generated by the infected host. Even after the recruitment of immune system cells, such as monocytes, macrophages, and neutrophils, the parasites develop the ability to evade the immune system, modifying the host's innate and adaptive immunity to promote its own survival [4–6]. The innate immune response generated after the first hours of infection is crucial for the establishment of the parasites in the intracellular environment of the host cell, either for cure or for parasitic persistence [7].

Inside the macrophages, the initial challenge of the parasite is to ensure its survival against the mechanisms of protection of those cells. After activation of the cells of the innate immune system, the development of the adaptive immune response occurs with the production of proinflammatory cytokines. This phase is polarized by two types of response: the first is Th1 response with the production of interferon gamma (IFN-*γ*) and tumour necrosis factor alpha (TNF-*α*), which leads to the activation of the oxidative stress machinery; still protecting the host cell against the parasite, it may induce a Th2 type response with the production of, mainly, interleukin 4 (IL-4) that leads to the phenotype of susceptibility to infection [8]. Another cytokine of the Th2 profile, also with regulatory function, the interleukin 10 (IL-10), is associated with the parasite persistence and resistance to the treatment in *Leishmania* infections, both in humans and in mice [9].

The drugs available with leishmanicidal action are limited, show high toxicity, and are poorly tolerated by the patients [10]. The search for alternative therapies is essential for the control of *L. amazonensis* infection, mainly because of the side effects and the high costs of the treatments currently used [11]. Based on these facts, the search for new therapeutic molecules is justified, and the use of endophytic microorganisms (bacteria and fungi that inhabit the interior of plants without causing damage to the host) [12] is a promising path. One of the advantages of endophytic utilization is associated with its metabolic capacity to produce a great diversity of antimicrobial peptides (PAMs) [13], and thus the endophytic *P. polymyxa* RNC-D, isolated from the cerrado of São Carlos (SP, Brazil), was chosen. Until now, no data were found in the literature on the action of the extract produced by the bacteria *P. polymyxa* RNC-D against the promastigote and amastigote forms of *L. amazonensis*, one of the species responsible for cutaneous manifestations of the disease.

In a bioprospecting study, a bacterium was isolated from *Prunus* spp. and identified as *P. polymyxa*. The endophytic microorganism was identified genotypically, with 99.2% when compared with *P. polymyxa*, and was called *P. polymyxa* RNC-D [14]. *P. polymyxa* has a great biotechnological potential: for example, the production of fibrinolytic enzymes [15], hydrolases [16], amylase [17], and bioactivity against phytopathogenic fungi [18]. The extract is a sample consisting of a set of substances, which may present specific biological activity. The importance of the search for new applications of these bioactive compounds with potential antitumor, antioxidant, antimicrobial, and antiparasitic activities is evident [14]. Thus, the objective of the presented work was to evaluate the effect of the total lyophilized extract of the endophytic microorganism *P. polymyxa* RNC-D in the experimental model of cutaneous leishmaniasis *in vitro*, in an attempt to obtain information that would enable a new therapy against this disease, since the extract can modulate the immune response, contributing to an effective response in the control of CL.

## 2. Materials and Methods

### 2.1. Lyophilized Total Extract Produced by *Paenibacillus polymyxa* RNC-D

The extract was donated by Professor Cristina Paiva de Sousa from the Department of Morphology and Pathology, Laboratory of Microbiology and Biomolecules (LaMiB), Federal University of São Carlos, Brazil. The endophytic bacterium *P. polymyxa* RNC-D was isolated from leaves of *Prunus* spp., which were collected from Brazilian tropical savannah in São Carlos, SP, Brazil [19]. The endophytic population was isolated and identified, and the strains were characterized phenotypically and genotypically [14]. The extract was prepared from bioactive metabolites, which were excreted by activated bacteria in culture medium (ISP2). There was no bacterial lysis in the process of obtaining these metabolites. The extract was then lyophilized and purified, and the fractions were obtained by High Performance Liquid Chromatography (HPLC) [20]. The extract was diluted in DMEM (Dulbecco's Modified Eagle's Medium, Sigma-Aldrich, USA) for conducting the experiment.

### 2.2. Parasites

Promastigote forms of *L. amazonensis* (strain MHOM/BR/73/M2269) were donated by Professor Sergio de Albuquerque from the Faculty of Pharmaceutical Sciences of Ribeirão Preto, University of São Paulo (USP). The culture medium used was 199 (Sigma-Aldrich, USA) supplemented with 10% fetal bovine serum (FBS) (Vitrocell Embriolife, Brazil), 1% penicillin and streptomycin (Sigma-Aldrich, USA), and 10% of L-glutamine (Sigma-Aldrich, USA), with the culture being maintained in incubator at 25°C.

### 2.3. Culture of RAW 264.7 Macrophages

The lineage of murine RAW 264.7 macrophages was donated by Professor Otávio Thiemann, from the Institute of Physics of São Carlos (IFSC, USP II), and was cultivated in DMEM (Sigma-Aldrich, USA) supplemented with 10% of inactivated FBS (Vitrocell Embriolife, Brazil), 1% of antibiotic mix penicillin and streptomycin (Sigma-Aldrich, USA), and 3.7 g/L of sodium bicarbonate (Sigma-Aldrich, USA). To perform the experiments, the cells were kept in growing bottles, and mechanical action with the aid of “cell scraper” (TPP®) was used for the detachment of the cell carpet of the bottles. The cell suspension was distributed in new vials of sterile culture and kept in a CO_2_ incubator (5%) at 37°C.

### 2.4. Viability of *Leishmania amazonensis* Promastigotes through the MTT Method

Cells viability was evaluated using the MTT colorimetric method (MTT [3-(4, 5-dimethylthiazol-2-yl)-2,5-diphenyltetrazolium bromide], Sigma-Aldrich, USA) [21]. In plates of 96 wells, 100 *μ*L of 1 × 10^7^/well of promastigotes in stationary phase was added with different concentrations of the extract (0.1, 0.5, and 1 mg/mL). As negative control, only culture medium was added, and, as positive control, 0.1 mM Amphotericin B/well (Sigma-Aldrich, USA) was added. After 24 and 48 hours of treatment, 100 *μ*L/well of a solution with 0.5 mg/mL of MTT was added and diluted in saline phosphate buffer (PBS, Sigma-Aldrich, USA) and incomplete culture medium. The plates were incubated at 25°C under protection from light for 4 hours. For the solubilization of the formazan crystals, 100 *μ*L of absolute isopropyl alcohol was added to each well. The absorbance reading was performed at 550 nm in a plate spectrophotometer (TP Reader NM, Thermo Plate, Nanshan District, Shenzhen, China). The cell viability was obtained in percentage and calculated with the absorbance of untreated cells (negative control) representing 100% of the cell viability. The value of a half-maximal effective concentration (EC_50_) was obtained from the values of the cytotoxic concentration and calculated from the percentage of viable cells. Three independent experiments were carried out.

### 2.5. Infection of RAW 264.7 Macrophages with *Leishmania amazonensis* and Posterior Treatment with *Paenibacillus polymyxa* RNC-D Extract

To perform the experiment, 500 *μ*L of culture medium containing 1 × 10^5^ cells/well was added in plates of 24 wells previously occupied with sterile glass coverslips and incubated in CO_2_ (5%) at 37°C for 24 hours for adhesion. Subsequently, the supernatant was discarded, and in each well 4 × 10^5^ (4 parasites/cell) promastigotes (in stationary phase) were added in a volume of 500 *μ*L of culture medium. Then, the plates were centrifuged at 3,000 rpm/5 min/25°C and taken to the incubator of CO_2_ (5%), at 37°C for 4 hours (time for parasite phagocytosis). After that, 500 *μ*L of different concentrations of the extract (0.1, 0.5, and 1 mg/mL) were added to the wells; for the negative and positive controls, culture medium and 0.1 mM of Amphotericin B were added, respectively. The plates were taken to the incubator under the same conditions as before for 24 and 48 hours. After the established periods, the supernatant was removed with a pipette and the wells were washed with 500 *μ*L PBS 1*X* at room temperature in order to withdraw possible extracellular parasites. Then, 10 *μ*L of SFB was added for the fixation of the slides that were subsequently stained with 250 *μ*L of Fast Panotic (Laborclin, Rio de Janeiro, Brazil). The plates were washed with 500 *μ*L of water three times, and the glass coverslips were removed from the wells for drying. For the fixation of the coverslips on microscopy slides, 10 *μ*L of Entellan was used (Merck, USA); after drying, the slides were taken to the microscope Olympus BX41 (immersion objective 100*x*) for capturing the images. For cell and amastigote count, the program ImageJ version 1.51 U was used. One hundred macrophages were counted in three independents experiments. The infection index was obtained by multiplying the percentage of cells infected by the mean number of parasites by infected cells, and the differences between the control, treated, and untreated cells were subsequently observed.

### 2.6. Evaluation of NO Production by RAW 264.7 Macrophages after Infection with *Leishmania amazonensis* against *Paenibacillus polymyxa* RNC-D Extract

To measure the production of NO, 1 × 10^5^ cells/well in 200 *μ*L were added in plates of 96 wells and incubated in a CO_2_ incubator for 24 hours for adhesion. The supernatant was removed, and the culture medium was added containing the treatment at different concentrations (0.1, 0.5, and 1 mg/mL) of the extract. As positive control, 100 ng/mL lipopolysaccharide (LPS) (Sigma-Aldrich, USA) was added, and, for negative control, only culture medium was used. For determination of nitrite derived from NO in macrophages infected with promastigotes, a volume of 500 *μ*L of culture medium containing 1 × 10^5^ cells/well was plated in plates of 24 wells previously occupied with sterile glass coverslips. They remained under the same conditions and were kept in a CO_2_ incubator for 24 hours. After adhesion, 4 × 10^5^ parasites (4 parasites/cell) were added to the plates and taken to incubator for 24 hours. Then, the plates were washed for removal of parasites that were not phagocytosed. Then, for the treatment, different concentrations (0.1, 0.5, and 1 mg/mL) of the extract were used, with their respective controls and with 0.1 mM Amphotericin B. NO quantification was performed in periods of 24 and 48 hours after infection. After the stipulated times, a volume of 100 *μ*L of the supernatant (of the treated cells and controls) was removed, and 100 *μ*L of the Griess reagent was added to it [22]. NO production was estimated by quantification of the stable NO metabolite, nitrite (NO_2−_). The absorbance at 550 nm was measured in a plate spectrophotometer (TP Reader NM, Thermo Plate, Nanshan District, Shenzhen, China) after 15 minutes. The nitrite concentration in the supernatant was quantified from a standard curve with known concentrations of sodium nitrite in *μ*M.

### 2.7. Quantification of Cytokines by Immunoenzymatic Assay (ELISA)

For the quantification of TNF-*α*, IFN-*γ*, IL-10, IL-4, and IL-12, we used the supernatants of RAW 264.7 macrophages infected with *L. amazonensis* and exposed to concentrations of 0.1, 0.5, and 1 mg/mL of the extract, after 24 and 48 hours with their respective controls. The procedure was performed according to the manufacturer's protocol (BD Biosciences®). The absorbance was obtained in the wavelength of 450 nm in a plate spectrophotometer reader (TP Reader NM, Thermo Plate, Nanshan District, Shenzhen, China). The solutions and the dilutions factor were performed according to the kit of each cytokine. The analysis was performed from the titration curve of the cytokine patterns, and the final concentrations were determined in pg/mL.

### 2.8. Statistical Analysis

The results were analyzed using the program Prism, version 5, GraphPad (2005) (San Diego, California, USA). The Kolmogorov–Smirnov Normality Test was applied for all data obtained. Subsequently, the one-way ANOVA (one-way analysis of variance) test was used for parametric data, and the post hoc test was performed using the Tukey method (Tukey's Multiple Comparison Test). For nonparametric data, the Kruskal–Wallis test and the post hoc Dunn's Multiple Comparison Test were performed. The statistical significance was established in values < 0.05. In order to calculate the values of EC_50_, nonlinear regressions of the averages of the values found for each concentration were made in at least three independent experiments.

## 3. Results

### 3.1. Evaluation of Leishmanicidal Activity of *Paenibacillus polymyxa* RNC-D Extract on Promastigotes of *Leishmania amazonensis*

**Figure 1:**
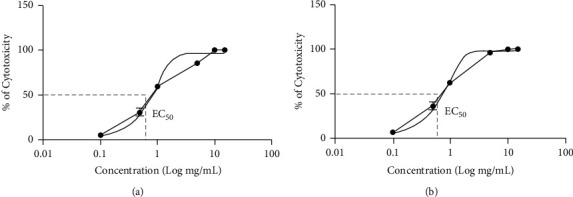
Leishmanicidal concentration-response graphs (%) of *Leishmania amazonensis* promastigotes exposed to different concentrations of *Paenibacillus polymyxa* RNC-D extract (0.1, 0.5, 1, 5, 10, and 15 mg/mL) at 24 and 48 hours. The figure shows EC_50_ values obtained from GraphPad Prism 5 software, with 95% confidence intervals. Results were obtained by MTT assay.

The extract toxicity was tested in promastigote forms of *Leishmania amazonensis* in the stationary phase, for 24 and 48 hours at different concentrations (0.1, 0.5, 1, 5, 10, and 15 mg/mL) (Figure 1). The ability of viable cells to reduce the MTT salt was assessed by the formation of formazan crystals which, subsequently solubilized, provided the absorbance values of the tests. The results obtained were compared to the negative control, which determined the EC_50_ in the analyzed periods. The calculation for the EC_50_ values was done with GraphPad Prism 5 software, with confidence intervals of 95%. EC_50_ values obtained for the 24- and 48-hour periods were 0.624 mg/mL and 0.547 mg/mL, respectively (Table 1).

### 3.2. Evaluation of Leishmanicidal Activity in Macrophages Infected with *Leishmania amazonensis* and Subsequent Treatment with *Paenibacillus polymyxa* RNC-D Extract

RAW 264.7 macrophages were cultured on coverslips, infected with *Leishmania amazonensis* promastigotes, and treated with different concentrations of *Paenibacillus polymyxa* RNC-D extract (0.1, 0.5, and 1 mg/mL); 0.1 mM Amphotericin B was used as positive control. The coverslips were fixed and stained for analysis. Through several images it was possible to perform the counting of the amastigote forms as shown in Tables 2 and 3.

### 3.3. Production of TNF-*α*, IFN-*γ*, IL-12, IL-4, and IL-10

**Figure 2:**
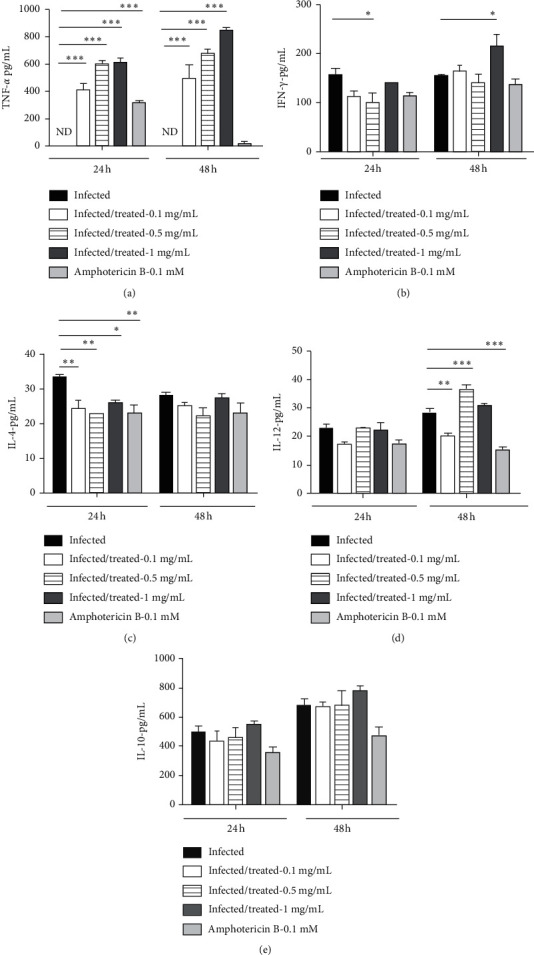
Detection of cytokines (TNF-*α*, IFN-*γ*, IL-12, IL-4, and IL-10) after infection with *Leishmania amazonensis* and posterior treatment with *Paenibacillus polymyxa* RNC-D extract. The symbols ^*∗*^*p* < 0.05, ^*∗∗*^*p* < 0.01, and ^*∗∗∗*^*p* < 0.001 represent the significant difference between the group exposed to the extract and the infected group (untreated) in periods of 24 and 48 hours. ND: not detected.

Figure 2 shows the production of TNF-*α*, IFN-*γ*, IL-12, IL-4, and IL-10 concentrations by RAW 264.7 macrophages in 24 and 48 hours after infection with *Leishmania amazonensis* and the subsequent treatment with different concentrations of the extract (0.1, 0.5, 1, 5, 10, and 15 mg/mL). A significant increase of TNF-*α*, IFN-*γ*, and IL-4 (Figures 2(a)–2(c)) was observed in the period of 24 hours, while in the period of 48 hours, it was observed an increase in the levels of IL-12 and IL-10 (Figures 2(a), 2(b), 2(d), and 2(e)).

### 3.4. Production of Nitric Oxide (NO) by RAW 264.7 Macrophages

**Figure 3:**
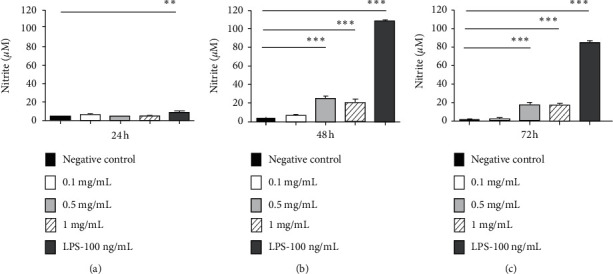
Quantification of NO produced by RAW 264.7 macrophages, after treatment with *Paenibacillus polymyxa* RNC-D extract.

Figure 3 and Table 4 show the production of NO by RAW 264.7 macrophages exposed to different concentrations of the extract (0.1, 1, and 0.5 mg/mL), as well as the control cells in the periods of 24, 48, and 72 hours. As negative and positive controls, culture medium and 100 ng/mL of LPS were used, respectively. The data presented in Figure 3 are also shown numerically in Table 4.

### 3.5. Production of NO by RAW 264.7 Macrophages during Infection with *Leishmania amazonensis* against *Paenibacillus polymyxa* RNC-D Extract

**Figure 4:**
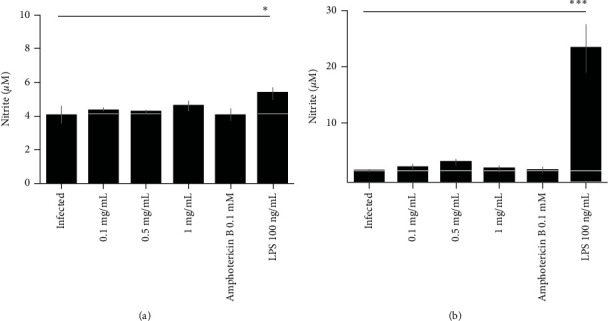
Quantification of NO produced by RAW 264.7 macrophages after infection with *Leishmania amazonensis* and subsequent treatment with *Paenibacillus polymyxa* RNC-D extract.

In order to evaluate the effects of different concentrations of the extract (0.1, 0.5, and 1 mg/mL) on the interaction between *Leishmania amazonensis* and host cells, the quantification of NO production in cell cocultivation supernatant (parasite + cell) was determined in the periods of 24 and 48 hours. As positive control, 0.1 mM of Amphotericin B was used; as negative control, only culture medium was used. The data presented in Figure 4 are also shown numerically in Table 5.

## 4. Discussion

Treatment of CL continues to be a health concern, and alternative therapies with fewer side effects are substantially needed [23]. The bioprospection of new compounds that present low EC_50_ values provides lower chances of undesirable effects in the development of a new drug. Our results showed that the values of EC_50_ obtained in the periods of 24 and 48 hours of leishmanicidal were 0.624 mg/mL and 0.547 mg/mL, respectively (Table 1). Neris and collaborators [24] have previously observed the effect of the *Paenibacillus polymyxa* RNC-D extract in BALB/3T3 fibroblasts and J774A.1 macrophages. In the present study, it was reported that the mortality rate of 50% was around 1.171 mg/mL for fibroblasts and 0.994 mg/mL for macrophages in the period of 48 hours. These data show that the promastigote forms of *L. amazonensis* were more sensitive to the action of the extract than the tested cell lines since the necessary concentration for the death of 50% of the promastigotes causes the death of less than 50% of the cells reported in 48 hours. This disparity may have occurred because promastigotes have more than 60% of anionic lipophosphoglycan in the plasma membrane [25]. These molecules are negatively charged, and they attract the PAMs, since the latter are positively charged [26, 27]. It is known that the PAMs act directly on the plasma membrane, and this barrier is very important for the establishment and the acquisition of nutrients. Therefore, drugs that destabilize the surface of the plasmatic membrane in terms of its functions and integrity can be considered as good chemotherapies for the treatment of leishmaniasis [28].

The host-parasite relationship should always be considered when seeking new compounds for the treatment of diseases. Thus, the *in vitro* test of macrophages coinfected with *Leishmania* is an important parameter for the prospection of new compounds. Our data (Table 2) showed that the percentage of infected cells in the control group was 53% with an average of 4.5 amastigotes/cell. In the cells treated with 0.1, 0.5, and 1 mg/mL of the extract, a percentage of approximately 25% of infected cells with an average of 3 amastigotes/cells was observed. These data represent a reduction of 50% in infected cells when compared to the infected group (control). In the group treated with Amphotericin B, no infected cells were observed. A smaller number of viable macrophages (data not presented) were observed when compared to negative control and treatments. This fact can be explained by the cytotoxic action of Amphotericin B that may have contributed to the death of macrophages since this drug has toxic effects already known [29]. In the period of 48 hours, for the infected group, a decrease in infected cells (≈25%) was observed, with an average of 3 amastigotes/cell, when compared with the period of 24 hours. In the same period, in the cells treated with 0.5 and 1 mg/mL of the extract, the percentage of infected cells was 23%, with approximately 2 amastigotes/cell. In the period of 24 hours, the reduction of amastigotes was expressive in the cells treated with the extract, when compared to the untreated infected group, which was not observed in the period of 48 hours (Table 3). Macrophages infected with *L. amazonensis*, even in the presence of IFN-*γ* and TNF-*α*, fail to eliminate the parasites altogether due to the formation of the parasitic vacuole, which may compromise the leishmanicidal action of NO and ROS, favoring the maintenance of the infection [30–32].

The survival strategy used by these parasites can be the inhibition of hydrolytic enzymes and other destructive molecules that are secreted in phagolysosomes. Peroxiredoxins and superoxide dismutase degrade derivatives of nitrite and radical reactive oxygen intermediates, which are the most important microbicidal molecules [23, 31]. Nevertheless, as seen in Table 3, it was shown that the extract at the concentration of 1 mg/mL was able to reduce the number of infected cells in 50% in the period of 24 hours and induce the regulation of the microbicide activity of the analyzed macrophages.

The species *L. amazonensis* is known to inhibit the immune response of the host cell, interfering in the intracellular signaling pathways to avoid an effective immune response, thereby subverting the cellular machinery, and modulating the environment in its favor [33, 34].

It has been reported that macrophages infected with *L. amazonensis* produce less TNF-*α* even in the presence of IFN-*γ* [34, 35]. This suggests that the activation of macrophages infected with *L. amazonensis* is deficient *in vitro* [34, 35], and our findings corroborate this data. The nonproduction of TNF-*α* was observed in the group infected with *L. amazonensis* and untreated (Figure 2(a)), even with the detection of IFN-*γ* (Figure 2(b)). For all macrophages infected and treated with the extract, there was a significant increase in the production of TNF-*α* when compared with the infected group in the periods of 24 and 48 hours. A significant increase in the production of IFN-*γ* cytokine was observed in the period of 48 hours at the concentration of 1 mg/mL of the extract.

The nonsignificant detection of IFN-*γ* at concentrations of 0.1 and 0.5 mg/mL can be understood by the action of the IL-10 regulatory cytokines. For the group treated with Amphotericin B, there was significant production of TNF-*α* in the period of 24 hours. IFN-*γ* is essential in the activation of macrophages to fight against *Leishmania* [23]. This cytokine elevates the synthesis of the enzyme inducible nitric oxide synthase (iNOS) and toxic nitrogen reactive radicals for the parasite [32, 35, 36]. Our results suggested that the extract was able to induce significant TNF-*α* production in RAW 267 macrophages after infection with *L. amazonensis*. We consider these results to be relevant due to the macrophage being one of the first cells to be infected. Moreover, it could contribute to lesser avoidance of the parasite against the immune system.

It is known that IL-12 activates the production of IFN-*γ* and consequently the differentiation of T cells in Th1 [37]. This process leads to the production of IFN-*γ* which results in the production of NO and death of *Leishmania*. Thus, IL-12 participates in the beginning of a protective cellular response against the disease [37]. Our results showed that there was significant production of IL-12 in the infected group treated with 0.5 mg/mL (Figure 2(d)). For the other concentrations of the extract (0.1 and 1 mg/mL), there was no significant production of this cytokine, which may be the result of the regulation of IL-10, since significant production of NO was observed in the periods of 24 and 48 hours in macrophages exposed to the extract [24].

Research using several experimental models (such as genetically deficient mice) has questioned the participation of IL-4 in the progress of chronic lesions. *In vivo* studies have determined that IL-4 is essential for the development of Th2 cells during the initial stage of infection. This fact was shown using anti-IL-4 antibodies in BALB/C mice, which were infected with *L. major* at the beginning of the infection. These antibodies promoted the polarization to the Th2 response profile, providing expansion of Th1 cells, with consequent healing of the lesion [38]. In our *in vitro* findings with *L. amazonensis*, a significant decrease in IL-4 was observed in all infected macrophages treated with the extract in the period of 24 hours when compared with the untreated infected group. These results are relevant, since cytokines such as IL-4, IL-10, and IL-13 may promote replication and survival of the parasites [39, 40]. Regarding the IL-10 cytokine, the study of Kane and Mosser [41] showed that the interaction of *Leishmania* with receptors on the macrophage surface induced preferably the production of high levels of IL-10. This cytokine produced by the murine macrophages infected with *L. major* inhibited the activation of the cells and decreased the production of inflammatory cytokines [42]. This regulation often favors infection and compromises the efficacy of the immune system in eliminating parasites. In relation to the present study, the results showed that there was no significant production of IL-10 in all infected macrophages treated with the extract (Figure 2(e)), which may have occurred due to a modulation in the Th1/Th2 response in this experimental model.

It was found that, during the period of 24 hours, there was a significant increase of NO in the group exposed to LPS when compared with the negative control group (without treatment). In the period of 48 and 72 hours, there was a significant increase of NO in the group treated with 0.5 mg/mL and 1 mg/mL of the extract and also in the group exposed to LPS, when compared with the negative control (Table 4). This suggests that the extract, though presenting a lower potency, was also able to induce macrophages to produce NO when compared to LPS. Another observed finding is that the production of NO induced by the extract was dependent on time and concentration, and it showed important production at concentrations of 0.5 and 1 mg/mL in 48 hours. It is known that NO is induced during macrophage activation and contributes to the control of replication or neutralization of intracellular pathogens, as in the case of *Leishmania* [43]. Thus, our results suggest that the extract used seems to modulate positively the microbicidal activity of macrophages, one of the most important cells in *Leishmania* infection. These data can contribute to the induction of immunotherapeutic effect in the control of leishmaniasis.

A study similar to ours showed that the *ß*-Glucan compound produced by *P. polymyxa* JB115 induces the production of NO in RAW 264.7 macrophages, being also dependent on concentration and time [44]. Thus, based on our findings, we suggest that our extract has potential as an immunostimulant or even as an adjuvant for vaccines. However, further studies should be performed in order to validate these hypotheses.

The infected group treated with LPS produced significantly more NO when compared to the infected group without treatment, in the periods of 24 and 48 hours. It is possible to observe that, in the previous results (Table 5), the extract was able to induce significant NO production in macrophages in the period of 48 hours. The nonproduction of NO due to infection and treatment with the extract in the period of 48 hours can be explained, highlighting that some species of *Leishmania* possess the ability to resist the action of the macrophage's oxidative “burst,” apparently silting mechanisms of NO synthesis and reactive oxygen species [44].

## 5. Conclusions

The extract of *P. polymyxa* RNC-D promoted a leishmanicidal effect at the concentrations of 0.5 and 1 mg/mL, and it was able to modulate the production of cytokines TNF-*α*, IFN-*γ*, IL-12, and IL-4 by RAW 264.7 macrophages infected with *L. amazonensis*. In addition, the extract promoted the production of NO in RAW 264.7 macrophages at the concentrations of 0.5 and 1 mg/mL. These results show that the analyzed extract may be a potential candidate for the development of new drugs against leishmaniasis, since it modulates the immune response, contributing to an effective response in the control of cutaneous leishmaniasis.

## Figures and Tables

**Table 1:** EC50 values for periods of 24 and 48 hours in promastigotes of *Leishmania amazonensis*.

Exposure (h)	EC_50_ (mg/mL)	95% confidence intervals
24	0.624	0.503–0.745
48	0.547	0.431–0.664

**Table 2:** Percentage of cells infected with *Leishmania amazonensis* and average amastigotes per cell after 24 hours of treatment.

Group	% Infected cells	Amastigotes/cell
Control	53 ± 2.5	4.5 ± 0.02
0.1 mg/mL	23 ± 5	2.5 ± 0.08
0.5 mg/mL	25 ± 5	3.3 ± 0.5
1 mg/mL	25 ± 4	3.2 ± 1.07
Amphotericin B 0.1 mM	0	0

**Table 3:** Percentage of cells infected with *Leishmania amazonensis* and mean amastigotes per cell after 48 hours of treatment.

Group	% Infected cells	Amastigotes/cell
Control	25 ± 4.5	3.2 ± 1.1
0.1 mg/mL	22 ± 5.5	2.2 ± 0.4
0.5 mg/mL	23 ± 5	2.3 ± 0.5
1 mg/mL	23 ± 0	2.2 ± 0
Amphotericin B 0.1 mM	0	0

**Table 4:** NO concentration in the cells treated and not treated with *Paenibacillus polymyxa* RNC-D extract.

Group	24 h (*µ*M)	48 h (*µ*M)	72 h (*µ*M)
Control	4.216 ± 0.1^NS^	4.632 ± 0.2^NS^	2.118 ± 0.5^NS^
0.1 mg/mL	5.835 ± 1.5^NS^	6.428 ± 1.7^NS^	2.519 ± 0.6^NS^
0.5 mg/mL	4.726 ± 0.5^NS^	24.936 ±.5^*∗∗∗*^	17.411 ± 4.6^*∗∗∗*^
1 mg/mL	4.868 ± 1.2^NS^	20.636 ± 6.0^*∗∗∗*^	17.353 ± 3.4^*∗∗∗*^
LPS-100 ng/mL	8.724 ± 2.5^*∗∗∗*^	108.385 ± 2.4^*∗∗∗*^	84.678 ± 4.7^*∗∗∗*^

^*∗∗*^ *p* < 0.01 and ^*∗∗∗*^*p* < 0.001 represent the significant difference between the treated group and the control group (no treatment). NS: without statistical difference.

**Table 5:** NO concentration in the cells treated and not treated with *Paenibacillus polymyxa* RNC-D extract after infection with *Leishmania amazonensis*.

Group	24 h (*µ*M)	48 h (*µ*M)
Control	4.094 ± 0.684^**NS**^	2.907 ± 0.138^**NS**^
0.1 mg/mL	4.353 ± 0.159^**NS**^	3.438 ± 0.665^**NS**^
0.5 mg/mL	4.268 ± 0.104^**NS**^	4.394 ± 0.481^**NS**^
1 mg/mL	4.616 ± 0.352^**NS**^	3.253 ± 0.542^**NS**^
Amphotericin B 0.1 mM	3.585 ± 0.511^**NS**^	3.137 ± 0.436^**NS**^

The symbols ^*∗∗*^*p* < 0.01 and ^*∗∗∗*^*p* < 0.001 represent the significant difference between the treated group and the negative control group (only infected). NS: no statistical difference.

## Data Availability

The data used to support the findings of this study are included within the article.
